# P2X7 receptor: A receptor closely linked with sepsis-associated encephalopathy

**DOI:** 10.1515/biol-2022-0775

**Published:** 2024-04-05

**Authors:** Zhao Fan, Kaifang Wang, Xiaoyong Zhao, Xude Sun

**Affiliations:** Shandong Provincial Medicine and Health Key Laboratory of Clinical Anesthesia, School of Anesthesiology, Weifang Medical University, Weifang 261053, Shandong, China; The Affiliated Hospital of Weifang Medical University, Weifang 261021, Shandong, China; Department of Anesthesiology, Tangdu Hospital, Air Force Military Medical University, Xian 710038, Shanxi, China

**Keywords:** P2X7 receptor, microglial cells, apoptosis, blood–brain barrier, sepsis-associated encephalopathy

## Abstract

Sepsis is defined as a dysregulated host response to infection resulting in life-threatening organ dysfunction. Sepsis-associated encephalopathy (SAE) is the main manifestation of sepsis. Inflammation, peroxidation stress injury, and apoptosis are the main factors involved in the pathogenesis of SAE. A growing body of evidence has proved that P2X7 receptor (P2X7R), a cationic channel receptor that is widely distributed in the body, plays a major role in the occurrence and development of inflammatory injury. Therefore, this review mainly describes the activation of P2X7R in sepsis, which leads to the recruitment of inflammatory cells to the cerebral vasculature, the destruction of the blood–brain barrier, the activation of microglial cells in the brain, the apoptosis of brain cells, and other damage processes. This review also illustrates the potential therapeutic value of P2X7R inhibition in SAE.

## 1 Introduction

Sepsis-associated encephalopathy (SAE) is a common central nervous system injury syndrome caused by sepsis. SAE is defined as diffuse brain damage secondary to infection in other parts of the body, without any signs of direct infection in the brain [1]. Although the pathogenesis of SAE is complex and diverse, peripheral infection is always the initial factor [2]. The key pathological changes in the brain that induce encephalopathy in patients with sepsis include the destruction of the blood–brain barrier (BBB), autophagy of cells, activation of microglial cells, and release of numerous inflammatory factors. Some clinical studies have found that complication with encephalopathy is an important cause of sudden death in most sepsis patients admitted to the intensive care unit [3]. Moreover, of the few SAE patients who do survive, most develop near-term or long-term cognitive impairment [4].

The human *P2X7* gene encodes the 595-amino acid protein P2X7R comprising a bulky extracellular domain and N- and C-terminal residues that are both on the cytoplasmic side. P2X7R is a purinergic ion channel receptor that is activated by ATP. As an ionic receptor, P2X7R, when activated, mainly induces a large influx of Ca^2+^ into the cytoplasm [5,6]. Studies have shown that P2X7R is highly expressed in brain cells, especially in the microglia and astrocytes [7,8]. When cells are stimulated by external stressors, the cell metabolism is enhanced, leading to the production of a large amount of ATP, which acts as a purinergic substance that has a strong activating effect on P2X7R; in SAE, lipopolysaccharides can reduce the activation threshold of P2X7R, and high concentrations of ATP cause the opening of P2X7R, leading to the profound perturbation of intracellular ion homeostasis [5]. The intracellular Ca^2+^ overload induced by P2X7R activation can cause a series of cell-damaging effects, such as mitochondrial damage, increased reactive oxygen species (ROS) production, and activation of the intracellular NLRP3 inflammasome, which then promote the activation of caspase-1 and the splicing of pro-interleukin (IL)-1β and pro-IL-18 into their active forms, IL-1β and IL-18 [9,10]. Alternatively, mitochondrial cytochrome C may be released into the cytoplasm, leading to apoptosis [11,12]. In microglial cells, P2X7R activation increases the production of the inflammatory cytokines IL-1β, IL-6, and tumor necrosis factor (TNF)-α [8]. Numerous studies have confirmed that P2X7R is highly expressed on the surface of glial cells in the brain [12]. Therefore, the present review aimed to characterize the specific molecular mechanism of P2X7R action in SAE and determine whether P2X7R inhibitors have therapeutic effects in SAE in order to gain insights into the pathogenesis of SAE and facilitate the development of new clinical treatment methods.

## 2 Effects of P2X7R on the BBB in sepsis

The BBB mainly consists of two parts: (a) the barrier between the plasma and the brain cells, which is mainly composed of brain capillary walls and glial cells and (b) the barrier between the plasma and the cerebrospinal fluid, which is mainly composed of the choroid plexus [13]. The gaps in the BBB are relatively small and less permeable, allowing only small molecules to pass through; hence, the BBB plays an important role in the protection of the internal environment of the brain [14].

**Figure 1:**
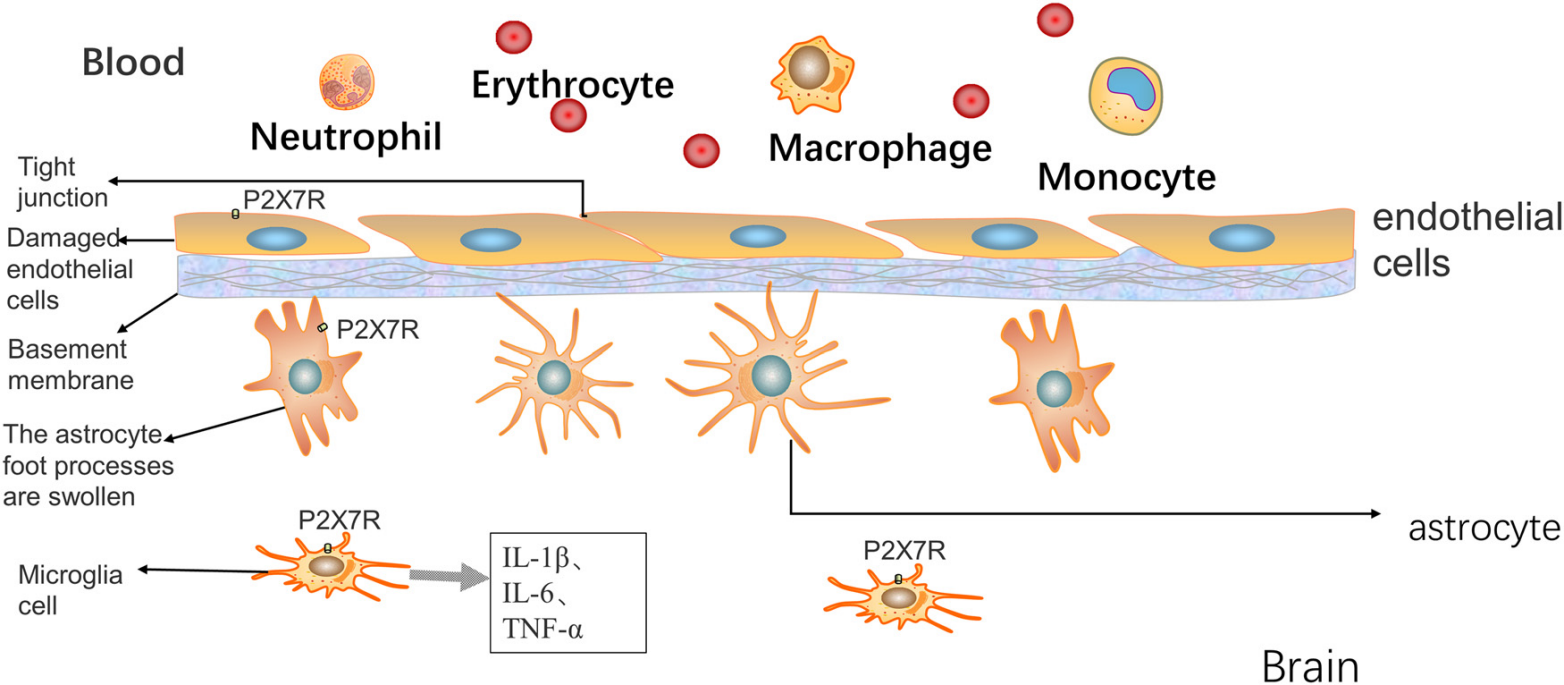
Sepsis induces leukocyte recruitment and damages the BBB. In severe sepsis, the stimulation of endothelial cells leads to the activation of surface P2X7R, causing self-injury and disruption of the tight intercellular junctions, followed by swelling and atrophy of the foot processes of the astrocytes below the basement membrane. Microglia activate and release inflammatory cytokines (IL-1β, IL-6, and TNF-α), which directly damage normal brain cells and cause brain dysfunction.

Sepsis causes damage to the BBB. We focused on the barrier between the plasma and the brain cells, which consists of capillary endothelial cells, basal membranes and their tight junctions, plus the foot processes of astrocytes [15,16]. Some studies have found that when the body is exposed to inflammatory mediators, such as lipopolysaccharides and bacteria, the white blood cells in the blood vessels produce a copious amount of harmful inflammatory mediators, which can lead to atrophy, membrane destruction, and even apoptosis of the cerebrovascular endothelial cells [17,18]. Since the tight-junction proteins that make up the BBB are mainly produced by the endothelial cells, the above changes are followed by a decrease in the expression of tight-junction proteins between the endothelial cells and the basement membrane as well as within the endothelial cells. The relatively common tight-junction proteins include transmembrane proteins, such as occludin, claudin, and JAM-1, and cytoplasmic attachment proteins, such as ZO-1 [19]. In a sepsis model established using cerebrovascular endothelial cells, western blot and immunofluorescence analyses showed that P2X7R expression was significantly increased in endothelial cells, and the tight-junction proteins of endothelial cells were significantly defective; moreover, P2RX7 knockdown ameliorated leukocyte-endothelial cell adhesion in septic mice [19]. In addition, trans-epithelial/trans-endothelial electrical resistance and fluorescein isothiocyanate-dextran 4 kDa trans-endothelial permeability assays have been used to evaluate the tight junctions of monolayer endothelial cells [20,21]. In mouse and rat sepsis models as well, the BBB permeability was significantly increased [22]. Under electron microscopy, the foot processes of glial cells were markedly swollen and contracted (Figure 1) [23]. However, these changes were reversed after the administration of P2X7R inhibitors, and both endothelial-cell function and glial-cell foot processes were significantly improved [24,25]. In addition, P2X7R inhibition reduced ROS production and accumulation in human brain microvascular endothelial cells (HBMECs) *in vitro* and inhibited the apoptosis signaling pathway associated with the mitochondrial serine protease Omi/HtrA2 in HBMECs *in vitro* [24–27]. The above research indicates that SAE caused by BBB destruction could be ameliorated by the use of P2X7R inhibitors. However, the specific intracellular molecular mechanism of the lesions induced by P2X7R activation remains unclear. Current evidence suggests the involvement of an imbalance in intracellular calcium homeostasis and the entry of extracellular small molecules (Ca^2+^ and Na^+^ influx and K^+^ efflux) caused by the opening of P2X7R macropores [5]. Therefore, further exploration is needed.

## 3 Effects of P2X7R activation on the expression of cerebrovascular chemokines and adhesion molecules in sepsis

P2X7R is associated with inflammatory injury. During inflammation, numerous chemokines are produced in the vascular endothelium within inflamed body parts to recruit white blood cells to inhibit the inflammation. As sepsis is a systemic intravascular infectious disease, during sepsis, the endothelial cells in the cerebrovascular blood vessels also produce various chemokines, such as CCL2, CCL3, CCL5, CXCL1, CXCL2, CX3CL1, and CXCL8. Recently, it was found that the activation of P2X7R on cerebrovascular endothelial cells during systemic sepsis can promote chemokine release, and thereby recruit white blood cells to aggregate in the brain [25]. Cell adhesion molecules play an important role in leukocyte chemotaxis and aggregation. Studies have shown that P2X7R can enhance the expression of intercellular adhesion molecule-1 in endothelial cells during inflammation and promote the binding of leukocytes expressing Mac-1 (CD11b/CD18) to brain endothelial cells [28]. This action causes the chemokine CX3CL1 produced by endothelial cells to enter the brain, where it recruits and activates microglia. Through this process, P2X7R activation transmits inflammatory signals from the cerebrovascular vessels to the brain tissue. This process also explains why there is no obvious sign of infection in the brain in the early stages of sepsis, but there is nonetheless inflammatory damage [29].

The intracerebral microcirculation is a key channel for the introduction of external inflammation into the brain. Studies have found that neutrophils and monocytes make up the majority of leukocytes recruited to the intracerebral microvessels (Figure 1), and the recruitment of CCR2+ monocytes to these vessels has been shown to impact cognitive function in a mouse model of sepsis [30]. However, P2X7R inhibition can reduce chemokine production in the cerebral microvessels, which can greatly inhibit the recruitment of these cells. At present, few studies have investigated the cerebrovascular role of P2X7R in SAE, and whether differing degrees of P2X7R activation during inflammation have different effects on the brain. Therefore, studying this topic is of great significance for further exploring the pathogenesis of SAE.

## 4 Effects of P2X7R on inflammatory cells in the brain in sepsis

It is well-known that the main inflammatory cells in the brain are the microglia. Microglia are characterized by multiple synapses and plasticity, and play an important role in maintaining the normal physiological function of the central nervous system [31,32]. They not only have a phagocytic function but are also implicated in the occurrence and development of many brain degenerative diseases [32,33]. In SAE, the destruction of the BBB leads to the entry of external inflammatory mediators into the brain, causing the activation of microglial cells [34]. Microglial cells phagocytose damaged brain cells to induce apoptosis or death [35–37]. The massive release of inflammatory cytokines (IL-1β, IL-6, TNF-α) and chemokines in SAE [38] produces two main results: the inflammatory cytokines directly damage normal brain cells and cause brain dysfunction, while the chemokines recruit more inflammatory cells and further amplify the inflammatory effects [39]. Studies have shown that when microglia are stimulated by external stressors (such as lipopolysaccharides) and active oxygen free radicals, they can induce the synthesis of the nuclear transcription factor NF-κB and indirectly increase the transcription of cytokines [40,41], such as apoptosis factors, the death receptor Fas, the death ligand FasL, TNF receptor 1 [42,43], and NLRP3 inflammasome.

P2X7R is associated with microglia. Experimental studies have found that during SAE, P2X7R is highly expressed and activated on the surface of microglial cells [44]. Other studies have shown that P2X7R is crucial for the activation of microglial cells and the development of intracranial inflammation. ATP-P2X7 activation by sepsis involves IL-6 production and STAT-3 activation in the brain, and P2X7R blockade has been found to diminish STAT3 activation in the cerebral cortex and hippocampus [45,46]. Pharmacological inhibition or genetic deletion of P2X7R attenuates the production of IL-1, TNF-α, and IL-10 [47]. Furthermore, both *in vivo* and *in vitro* treatment with P2X7R inhibitors can reverse the damage caused by the microglial cells in the brain tissue in response to inflammatory stimulation [48]. Thus, treatment with P2X7R inhibitors greatly alleviates a series of subsequent neurologically damaging events caused by microglial cell activation [49]. However, some authors have found that P2X7R, as a type of macropore, is a harmful channel and requires an appropriate concentration of ATP to be continuously maintained outside the cell [50]. When external inflammatory signals are transmitted to the brain through the BBB, they activate the microglia, which produce inflammatory mediators that direct brain cells to produce large amounts of ATP under a stress state, which maintains the extracellular ATP concentration and further activates P2X7R, forming a vicious cycle. If the P2X7R link in this cycle is blocked, the activation of microglial cells will be inhibited to a certain extent; on the other hand, inhibition of P2X7R on the surface of other cells in the brain will also have a protective effect [51]. In summary, P2X7R activation is not only a result but also a cause of microglia activation.

## 5 P2X7R can lead to brain-cell apoptosis and pyroptosis and cognitive dysfunction in SAE

P2X7R is associated with apoptosis. P2X7R is a Ca^+^ channel receptor whose activation induces a massive influx of calcium and sodium ions, which greatly impacts the physiology and biochemistry of the cell [5]. In SAE, the P2X7R on the surface membranes of astrocytes and microglia in the brain is activated, leading to massive Ca^2+^ influx, which has a fatal effect on mitochondria [52–54]. First, the excessive calcium ions destroy the oxidative respiratory chain in the mitochondria, which causes the production and release of a large amount of ROS into the cytoplasm. The ROS oxidize the methionine residues of calmodulin, thereby overactivating calmodulin and promoting apoptosis. ROS also upregulate apoptosis-inducing factor and induce its translocation to the nucleus, leading to caspase-dependent apoptosis [55,56]. Second, mitochondrial injury releases a large number of apoptosis-related proteins from the mitochondria, such as cytochrome C, Smac, and Omi, which enter the cytoplasm and trigger the activation of apoptotic signaling pathways [57]. For example, in the presence of dATP, cytochrome c binds with apoptosis-related factor 1 to form a polymer that binds and activates caspase-9, which in turn activates caspase-3 to induce apoptosis [58]. CD39 can bind to extracellular ATP and hydrolyze it to AMP, and CD39 genetic knockout has been found to exacerbate sepsis-induced injury in experimental cells [46,59]. However, for SAE induced by severe systemic sepsis, there is little direct evidence that P2X7R activation is associated with the release of mitochondrial apoptosis-related proteins into the cytoplasm. Studies have shown that P2X7R activation can indeed damage the mitochondria [53], so a future research prospect is to investigate the link between P2X7R and mitochondrial apoptosis-signaling pathways.

**Figure 2:**
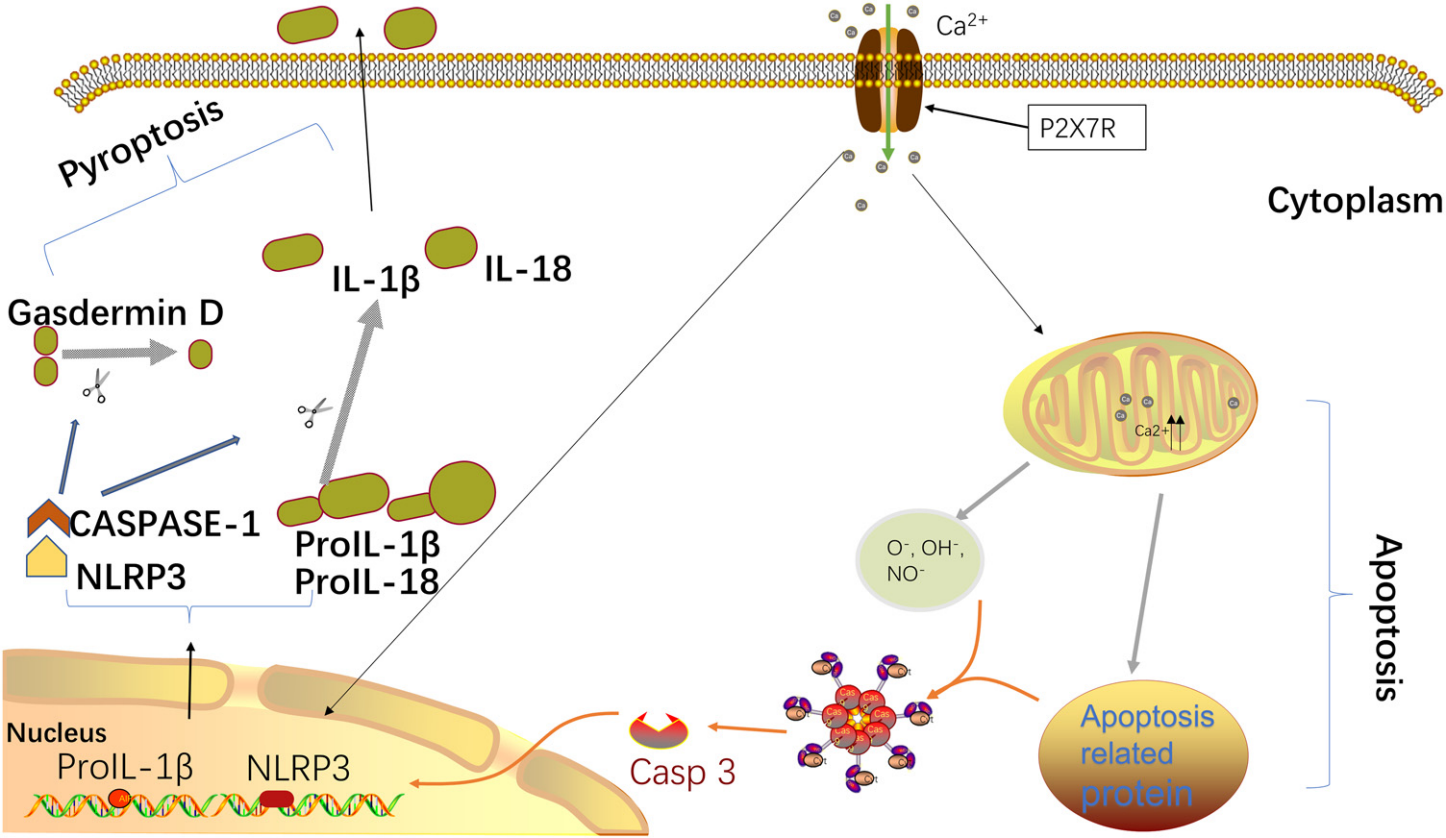
P2X7R activation induces apoptosis and pyroptosis. P2X7R activation causes calcium ion overload in the mitochondria and produces a large number of ROS and apoptosis-related proteins, which activate caspase-9, which in turn activates caspase-3 to induce apoptosis. Additionally, caspase-1 is activated through the NF-κB/NLRP3 pathway, and activated caspase-1 cleaves IL-1β, IL-18, and gasdermin D, causing pyroptosis.

P2X7R activation in sepsis induces the pyroptosis of microglial cells in the brain, leading to a series of functional disorders in the brain. Cell pyroptosis is also known as a type of inflammatory cell death [60]. Recent studies have found that cell pyroptosis occurs in many organs during sepsis. Inflammasomes play a crucial role in the classical pathway of cell pyroptosis [61]. Among the many different inflammasomes, NLRP3 is the most characteristic one. The basic structure of the NLRP3 inflammasome consists of NOD-like receptor (NLR) as the receptor protein, apoptosis-associated speck-like protein (ASC) as the adaptor protein, and caspase as the effector protein [62]. Studies in juvenile rats with sepsis have shown that P2X7R mediates NLRP3/caspase-1-related pyroptosis in the cerebral cortex through the ERK1/2 signaling pathway [63,64]. Under inflammatory stimulation, the activation of P2X7R on the surface of microglial cells will lead to the activation of intracellular NF-κB/NLRP3 signaling, and recruit and activate caspase-1 [65,66]. Caspase-1 cleaves and activates inflammatory factors such as IL-18 and IL-1β, and cleaves the *GSDMD* N-terminal sequence, which binds to the cell membrane to produce membrane pores, leading to cell pyroptosis (Figure 2) [67].

**Figure 3:**
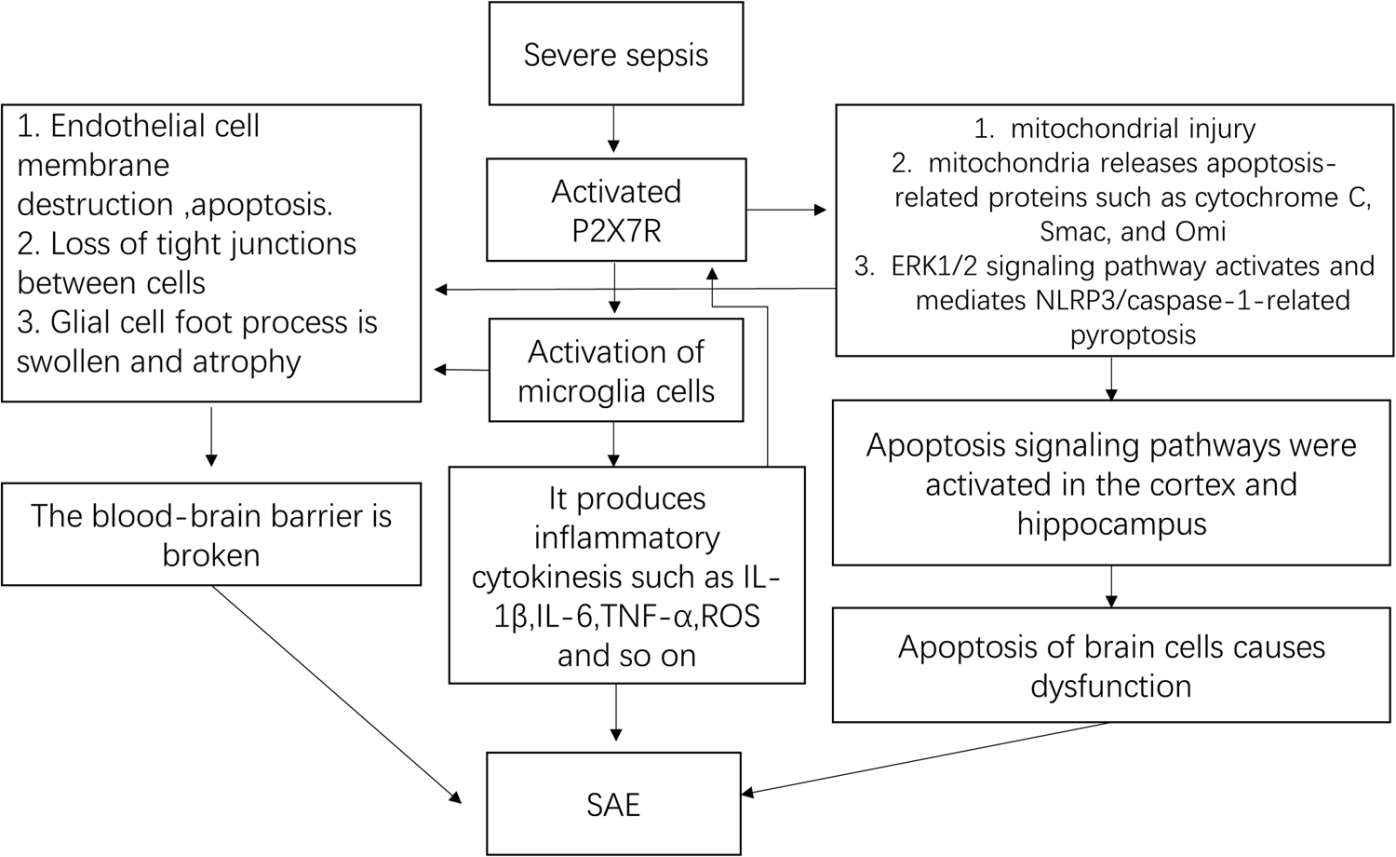
Mechanism of the induction of SAE by P2X7R activation in sepsis. Severe sepsis is the initial cause of SAE. Severe sepsis leads to the generation of a massive amount of inflammatory cytokines in the blood vessels. These cytokines stimulate endothelial cells to release a large amount of ATP, which activates P2X7R, thereby causing endothelial damage in the BBB. P2X7R activation also induces the activation of microglia, which produces more inflammatory cytokines, ROS, and NO. All these mediators subject the brain cells to strong stresses and cause more P2X7R activation, which leads to massive Ca^2+^ influx into the cell, causing mitochondrial damage and cell apoptosis.

P2X7R can improve cognitive function in mice. The areas of the brain that control cognitive function are concentrated in the hippocampus and cortical regions. Experimental studies have found that the damage inflicted by SAE is mainly concentrated in the cerebral cortex and hippocampus [29]. TUNEL and flow cytometry assays of the tissue in these two regions revealed a high incidence of apoptosis, which was reversed after the use of P2X7R inhibitors [68]. The main manifestation of SAE is the impairment of cognitive function, which has been verified by behavioral experiments in mice, and includes short-term or long-term memory loss, depression-like behavior, and the loss of exploration ability [69]. These changes have been linked to damage to the cortical and hippocampal cells in the brain. P2X7R inhibitors suppressed apoptosis and ROS production in the hippocampus and cortex, and reversed cognitive dysfunction in mouse models of SAE induced by lipopolysaccharides or caecal ligation and puncture (Figure 3) [68,70]. The above findings suggest that P2X7R has tremendous potential as a therapeutic target in SAE.

## 6 Conclusion

During severe sepsis, almost all organs suffer from oxidative stress injury, apoptosis, and pathological tissue necrosis caused by pyroptosis. This process is a good explanation for systemic inflammatory response syndrome. P2X7R is a purinergic ion channel receptor that is activated by ATP. During sepsis, the generation of a massive amount of inflammatory cytokines in the blood vessels leads to a large increase in ATP production, owing to which P2X7R is heavily activated, which ultimately leads to the activation of microglia. Microglia produce more inflammatory cytokines, ROS, and NO, causing more P2X7R activation. P2X7R activation causes calcium ion overload in the mitochondria and produces numerous ROS and apoptosis-related proteins, which activate caspase-9, which in turn activates caspase-3 to induce apoptosis. Additionally, caspase-1 is activated through the NF-κB/NLRP3 pathway, and activated caspase-1 cleaves IL-1β, IL-18, and gasdermin D, causing pyroptosis. Simultaneously, microglia activate and release inflammatory cytokines (IL-1β, IL-6, and TNF-α), which directly damage normal brain cells and cause brain dysfunction.

P2X7R plays an important role in SAE, and blocking P2X7R during the occurrence and development of sepsis can also reduce inflammatory responses and thereby reduce the damage caused to the heart, lungs, liver, and intestines [49,71–77]. In addition, P2X7R blockade alleviates cell lysis and pyroptosis [78]. However, the role of P2X7R in regulating vascular function depends on its location in both the macrovascular and microvascular endothelium, blocking P2X7R did not confer great protection in some vessels [37].Therefore, it is necessary to take this difference into account and investigate P2X7R-targeted therapy in the future.

Recent research on SAE has offered insights into its pathogenesis. Unlike degenerative brain diseases such as Alzheimer disease, SAE develops rapidly, and can occur at all ages. It also has a high mortality rate. Therefore, it is significant to find receptor targets that can be used to target the signaling pathways involved in the pathogenic mechanism of SAE. In this respect, the inhibition of P2X7R may be worth investigating, as P2X7R inhibitors have been shown to reverse both inflammation and apoptosis [68]. Current studies have mainly focused on the relationship between P2X7R and mitochondria-related apoptosis pathways, and have found that P2X7R activation in the brain cells during inflammation can cause the translocation of the apoptosis-related protein Omi from the mitochondria to the cytoplasm. Therefore, the relationship between Smac and P2X7R in the brain during sepsis warrants study. Furthermore, the development of drugs targeting P2X7R may have great potential for the prognosis and survival of SAE patients.
